# Construction and validation of an aging-related gene signature predicting the prognosis of pancreatic cancer

**DOI:** 10.3389/fgene.2023.1022265

**Published:** 2023-01-18

**Authors:** Dengchuan Wang, Yonggang Zhang, Xiaokang Wang, Limei Zhang, Shi Xu

**Affiliations:** ^1^ Office of Medical Ethics, Shenzhen Longhua District Central Hospital, Shenzhen, Guangdong, China; ^2^ Department of Clinical Laboratory, Shenzhen Longhua District Central Hospital, Shenzhen, Guangdong, China; ^3^ Department of Pharmacy, Shenzhen Longhua District Central Hospital, Shenzhen, Guangdong, China; ^4^ Department of Oncology, Shenzhen Longhua District Central Hospital, Shenzhen, Guangdong, China; ^5^ Department of Burn and Plastic Surgery, Shenzhen Longhua District Central Hospital, Shenzhen, Guangdong, China

**Keywords:** pancreatic cancer, aging, risk signature, TCGA, immune cell infiltration

## Abstract

**Background:** Pancreatic cancer is a malignancy with a high mortality rate and worse prognosis. Recently, public databases and bioinformatics tools make it easy to develop the prognostic risk model of pancreatic cancer, but the aging-related risk signature has not been reported. The present study aimed to identify an aging-related risk signature with potential prognostic value for pancreatic cancer patients.

**Method:** Gene expression profiling and human clinical information of pancreatic cancer were derived from The Cancer Genome Atlas database (TCGA). Aging-related gene sets were downloaded from The Molecular Signatures Database and aging-related genes were obtained from the Human Ageing Genomic Resources database. Firstly, Gene set enrichment analysis was carried out to investigate the role of aging process in pancreatic cancer. Secondly, differentially expressed genes and aging-related prognostic genes were screened on the basis of the overall survival information. Then, univariate COX and LASSO analysis were performed to establish an aging-related risk signature of pancreatic cancer patients. To facilitate clinical application, a nomogram was established to predict the survival rates of PCa patients. The correlations of risk score with clinical features and immune status were evaluated. Finally, potential therapeutic drugs were screened based on the connectivity map (Cmap) database and verified by molecular docking. For further validation, the protein levels of aging-related genes in normal and tumor tissues were detected in the Human Protein Atlas (HPA) database.

**Result:** The genes of pancreatic cancer were markedly enriched in several aging-associated signaling pathways. We identified 14 key aging-related genes related to prognosis from 9,020 differentially expressed genes and establish an aging-related risk signature. This risk model indicated a strong prognostic capability both in the training set of TCGA cohort and the validation set of PACA-CA cohort and GSE62452 cohort. A nomogram combining risk score and clinical variables was built, and calibration curve and Decision curve analysis (DCA) have proved that it has a good predictive value. Additionally, the risk score was tightly linked with tumor immune microenvironment, immune checkpoints and proinflammatory factors. Moreover, a candidate drug, BRD-A47144777, was screened and verified by molecular docking, indicating this drug has the potential to treat PCa. The protein expression levels of GSK3B, SERPINE1, TOP2A, FEN1 and HIC1 were consistent with our predicted results.

**Conclusion:** In conclusion, we identified an aging-related signature and nomogram with high prediction performance of survival and immune cell infiltration for pancreatic cancer. This signature might potentially help in providing personalized immunotherapy for patients with pancreatic cancer.

## 1 Introduction

Pancreatic cancer (PCa) is one of the highest mortality rate among all main cancer types and its incidence is increasing every year (Xu et al., 2020). In 2016, pancreatic cancer exceeded breast cancer and became the third leading cause of cancer death in the United States (Tempero, 2019). In the early stage, PCa is characterized by difficult diagnosis and occurs tumor invasion and metastasis (Ren et al., 2018; Tempero, 2019). Adjuvant chemotherapy after surgical resection is the primary treatment modality for early PCa (Zeng et al., 2019; Nie et al., 2021). In advanced stage, 5-fluorouracil/leucovorin with irinotecan and oxaliplatin and gemcitabine/nab-paclitaxel can largely improve the prognosis of PCa (Zeng et al., 2019). Nevertheless, the prognosis of PCa is still poor and the survival time of PCa patients is not significantly prolonged in the last decades (Kim et al., 2020; Nie et al., 2021). Hence, there is a need to identify new early diagnosis methods, prognostic risk factors and precision treatment.

According to numerous studies over the past decades, aging draws many parallels with cancer. At first glance, cancer and aging seem to be the opposite process: cancer is the result of abnormal enhancement of cell adaptability (Ullah et al., 2020) yet aging is marked by the gradual decline in tissue function and integrity (Venturelli et al., 2018). However, at a deeper level, cancer may have the same origin with aging. Aging and tumor share an important hallmark, that is, genomic stability (Hanahan and Weinberg, 2011; Lopez-Otin et al., 2013). Accumulation of DNA damage is the main cause of aging (Gems and Partridge, 2013). Similarly, DNA damage occasionally brings abnormal benefits to some cells, eventually leading to cancer (Kong et al., 2016). In a sense, they can be considered as two distinct manifestations of the same potential process, that is accumulated cell damage. Additionally, aging is an effective barrier to prevent tumorigenesis in cancer (Calcinotto et al., 2019). Thus, inducing senescence in cancer cells may also become a novel cancer treatment strategy.

The purpose of this study was to identify aging-related core genes in PCa, comprehensively characterize the impact of aging related core genes on tumor occurrence and development in tumor, and construct an aging-related gene pairs for predicting prognosis and therapeutic effect in PCa. To facilitate clinical application, a predictive nomogram was established to quantify the risk of death for individual PCa patients. In addition, Cmap database was used to screen potential candidate drugs for the treatment of high-risk PCa patients, and molecular docking analysis was used to validate. HPA database was used to verify the protein expression and survival analysis of aging related genes.

## 2 Materials and methods

### 2.1 Data acquisition

We obtained transcriptome profiling data and corresponding clinical data of PCa of TCGA and normal pancreas data of GTEx from UCSC Xena browser (https://xenabrowser.net/datapages/).The TCGA dataset was used as training set. The validation set for pancreatic tumors were collected from ICGC (PACA-CA) and GEO (GSE62452). Statistics of the sample size are shown in (Table 1). All the downloaded data in this study were standardized by the contributors. Fifty two aging-related gene sets were retrieved from The Molecular Signatures Database (MSigDB) (https://www.gsea-msigdb.org/gsea/index.jsp). Then, we collected 49 aging-related gene sets reported by Yue et al. (2021). Ultimately, we integrated the two gene sets to form 80 aging-related gene sets after removing overlapping genes. A total of 307 aging-related genes (ARGs) were obtained from the Human Ageing Genomic Resources database (HAGR) (https://genomics.senescence.info/).

**TABLE 1:** Clinical characteristics of PCa patients from TCGA, ICGC, and GEO.

Clinical variables	TCGA		PACA-CA		GSE62452	
	Number	%	Number	%	Number	%
Total	177	100	186	100	65	100
Age						
≤60 years	59	33.33	55	29.57	-	-
>60 years	118	66.67	112	60.22	-	-
unknown	0		19	10.22		
Gender						
Male	97	54.80	83	44.62	-	-
Female	80	45.20	102	54.84	-	-
unknown	0	0.00	1	0.54		
Stage						
Ⅰ	21	11.86	51	27.42	4	6.15
Ⅱ	146	82.49	86	46.24	44	67.69
Ⅲ	3	1.69	7	3.76	10	15.38
Ⅳ	4	2.26	0	0.00	6	9.23
unknown	3	1.69	42	22.58	1	1.54
Tumor classification						
T1	7	3.95	-	-	-	-
T2	24	13.56	-	-	-	-
T3	141	79.66	-	-	-	-
T4	3	1.69	-	-	-	-
Tx	2	1.13	-	-	-	-
History of diabetes						
Yes	38	21.47	-	-	-	-
No	108	61.02	-	-	-	-
unknown	31	17.51	-	-	-	-
History of smoking						
1 year	66	37.29	-	-	-	-
2 years	20	11.30	-	-	-	-
3 years	28	15.82	-	-	-	-
4 years	23	12.99	-	-	-	-
5 years	7	3.95	-	-	-	-
unknown	33	18.64	-	-	-	-
History of chronic pancreatitis			-	-	-	-
Yes	13	7.34	-	-	-	-
No	128	72.32	-	-	-	-
unknown	36	20.34	-	-	-	-
Survival time						
≤3 years	161	90.96	147	79.03	54	83.08
>3 years	16	9.04	39	20.97	11	16.92
Survival status						
alive	89	50.28	34	18.28	16	24.62
dead	88	49.72	152	81.72	49	75.38

### 2.2 Gene set enrichment analysis (GSEA)

GSEA is a computing methodology used to evaluate whether a given set of genes shows statistically significant between two biological states (Yoon et al., 2020). To study the role of aging in pancreatic cancer, GSEA analysis was completed using ClusterProfiler R package with parameters: nPerm = 1,000, *p*-value Cutoff = 0.05 and pAdjustMethod = “BH”.

### 2.3 Differential expression analysis

The differentially expressed genes (DEGs) between PCa and normal tissues were identified by using the R software package “limma”. Genes with |logFC|> = 1 and FDR < 0.01 were called significant. Volcano and heatmap plot were used to visualize the DEGs.

### 2.4 Construction of a prognostic aging-related signature

Univariate Cox regression analysis was applied to screen out the prognostic survival-related genes. The prognostic genes were displayed by a forest plot using “forestplot” in R. Then, the selected most significant prognostic survival-related genes were further screened and confirmed by the least absolute shrinkage and selection operator (LASSO). The risk score value of the aging-related gene signature for each sample was calculated using the following formula:

$${risk\;score=\sum }_{i=1}^{n}expi\beta i$$ (1)

(exp: Expression level, β: the regression coefficient).

All PCa patients were classified into a high-risk group and a low-risk group by the median risk score. Eventually, KM analysis was conducted to evaluate the differences in overall survival (OS) rates between two groups in the training and validation sets. The area under the curve (AUC) of the receiver operating characteristic (ROC) curve was calculated to examine the aging-related gene signature performance using the time ROC R package.

### 2.5 The clinical correlation analysis

We collected clinical indicators, including stage, gender, smoking, age, chronic pancreatitis, and diabetes of pancreatic cancer from TCGA, and compared the differences in survival indicators in different risk groups. A box plot was used to visualize the comparative analysis, and the Wilcoxon test was employed to calculate the significance *p*-value. *p* < 0.05 was used as the cutoff to screen out significant clinical features. Univariate and multivariate analyses of clinical variables and risk score were performed using Cox regression in training and validation cohorts.

### 2.6 Construction and evaluation of nomogram

We constructed a nomogram using the “rms” R package to estimate the 1-year, 3-years and 5-years survival rates of PCa patients based on PACA-CA dataset. The discrimination and accuracy of the nomogram was evaluated by the calibration curves. Moreover, DCA was used to identify the clinical application of the model.

### 2.7 The immune microenvironment correlation analysis

Immune and stromal cells are two major non-tumor components in the tumor microenvironment (TME), which are of great value in tumor diagnosis and prognosis evaluation. Immune and stromal scores were estimated the ratio of the immune and stromal components in TME by the ESTIMATE algorithm. The tumor purity of each sample was also compared between the different risk groups. At the same time, the Spearman correlation was used to test the correlation between immune score, stromal score, ESTIMATE score and tumor purity and risk score.

We calculated the proportion of infiltration levels of different immune cells between high and low risk score groups based on the following three methods: 1) The combination of CIBERSORT (https://cibersort.stanford.edu/) and LM22 (leukocyte signature matrix) can be used to evaluate the proportions of 22 human leukocyte cell subsets, 2) Single sample gene set enrichment analysis (ssGSEA) was applied to assess the proportions of 28 types of infiltrating immune cells of the tumor samples. 3) The xCell algorithm calculates infiltration of 64 immune cells.

### 2.8 Prediction of potential drugs for PCa treatment

Cmap is a gene expression profile database, mainly used to reveal the functional relationship between compounds, genes and disease status. The DEGs between low- and high-risk groups were identified and entered as query terms in the cMap database. Small molecular drugs with positive connectivity values were selected as potential therapeutic molecules for the treatment of PCa because of their antagonistic effects on aging-related genes’ expression. In this study, we screened the top ten small molecules and obtained their 2D molecular structures through the PubChem database (https://pubchem.ncbi.nlm.nih.gov/). At the same time, the protein structure of high-risk group core genes was obtained from the RCSB PDB database (PDB, http://www.pdb.org/). Autodock Vina-1.1.2 was used for molecular docking between candidate molecules and targets. The docking free energy < −5.0 kcal/mol was considered as stable binding. Finally, we used PyMOL-2.4.0 and LigPlot^+^v2.2 to visualize the molecular docking results.

### 2.9 Immunohistochemical analysis and survival analysis of ARGs

The expression of ARGs in normal and tumor tissues of our risk model was obtained from HPA database (https://www.proteinatlas.org/). At the same time, the survival analysis results of ARGs were also downloaded from this database.

### 2.10 Statistical analysis

In this study, all statistical analysis operations were carried out based on Rsoftware3.6.1. |logFC| > 1 and *p* < 0.05 were set as the screening criteria of DEGs. Wilcoxon rank sum test was conducted to compare gene expression of any two groups, while Kruskal–Wallis test was used for comparison among multiple groups. Hazard ratios were computed and independent prognostic factors were screened using the univariate and multivariate Cox proportional hazards regression models. Moreover, we determined the accuracy of our gene signature by generating ROC curves. For all statistical analyses, ns denotes not significant, * denotes *p* < 0.05, ** denotes *p* < 0.01, *** denotes *p* < 0.001, **** denotes *p* < 0.0001.

## 3 Results

### 3.1 The aging process participated in PCa progression

**FIGURE 1:**
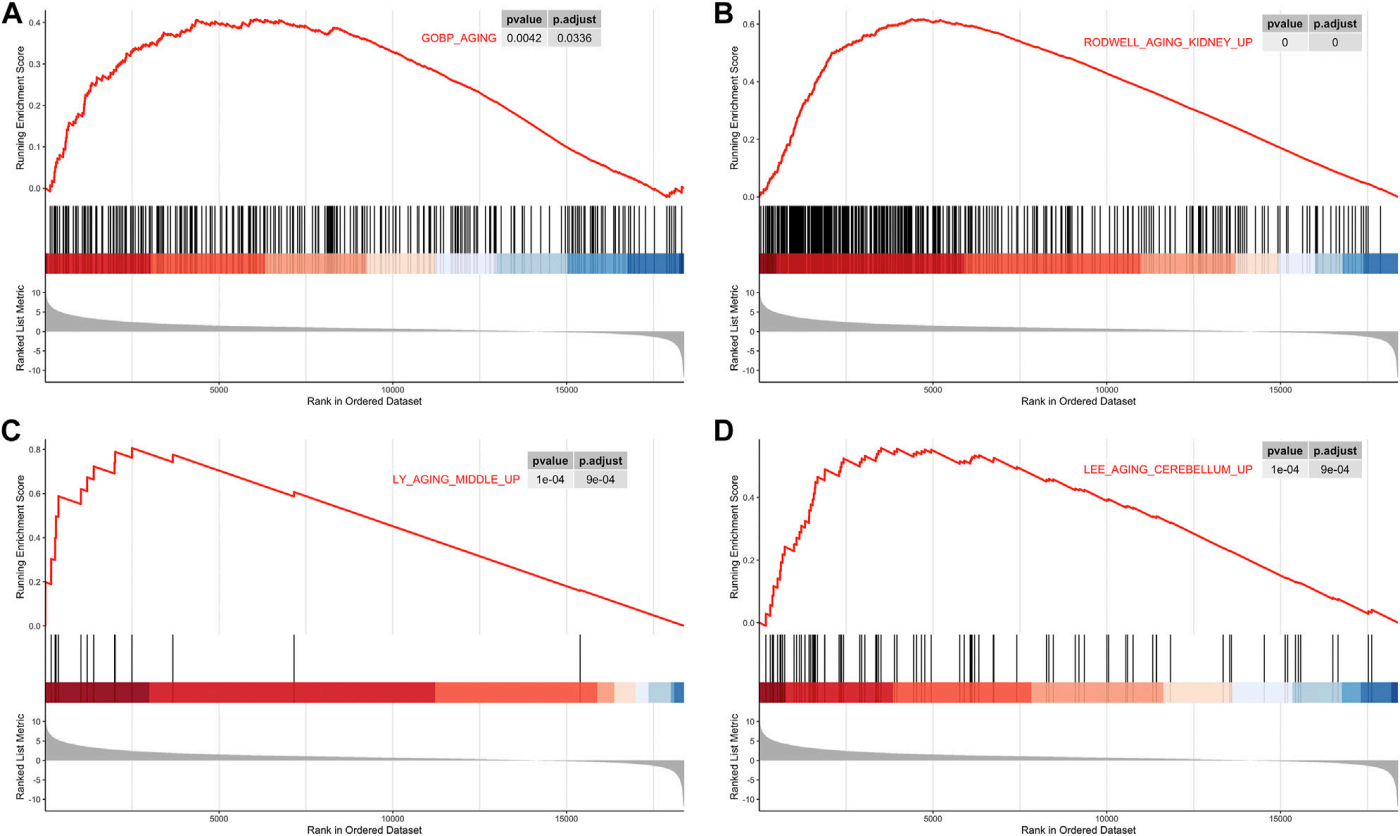
Gene Set Enrichment Analysis (GSEA). **(A)** GSEA analysis of the GOBP_AGING. **(B)** GSEA analysis of RODWELL_AGING_KIDNEY_UP. **(C)** GSEA analysis ofLY_AGING_MIDDLE_UP. **(D)** GSEA analysis of LEE_AGING_CEREBELLUM_UP.

Based on TCGA and GTEx datasets, we applied the R package limma to analyze the gene expression profile of tumor samples and control samples. All genes were sorted by logFC values. The ranked list of genes was then used to perform the GSEA analysis on the gene sets of “GO_AGING, RODWELL_AGING_KIDNEY_UP, LY_AGING_MIDDLE_Up and LEE_AGING_CEREBELLUM_UP ”. Compared with normal tissues, aging-related gene sets were distinctively activated in PCa tissues (Figure 1; Supplementary Figure S1).

### 3.2 Identification of DEGs and prognostic ARGs in the training group

**FIGURE 2:**
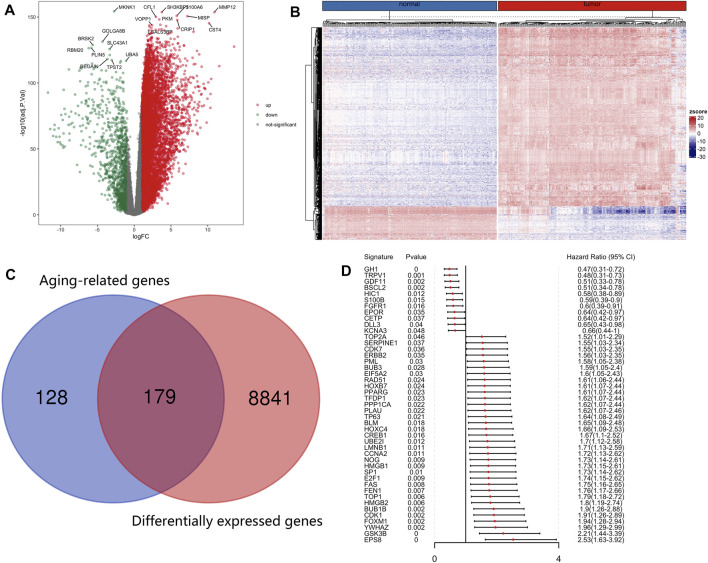
Identification of differentially expressed and prognostic aging-related genes in PCa. **(A)** Differentially expressed aging-related genes in the TCGA was displayed in the volcano map and **(B)** the heatmap. **(C)** The differentially expressed aging-related genes were screened by taking the intersection of DEGs and aging-related genes. **(D)**Forest plot of prognostic aging-related genes in the training group.

**FIGURE 3:**
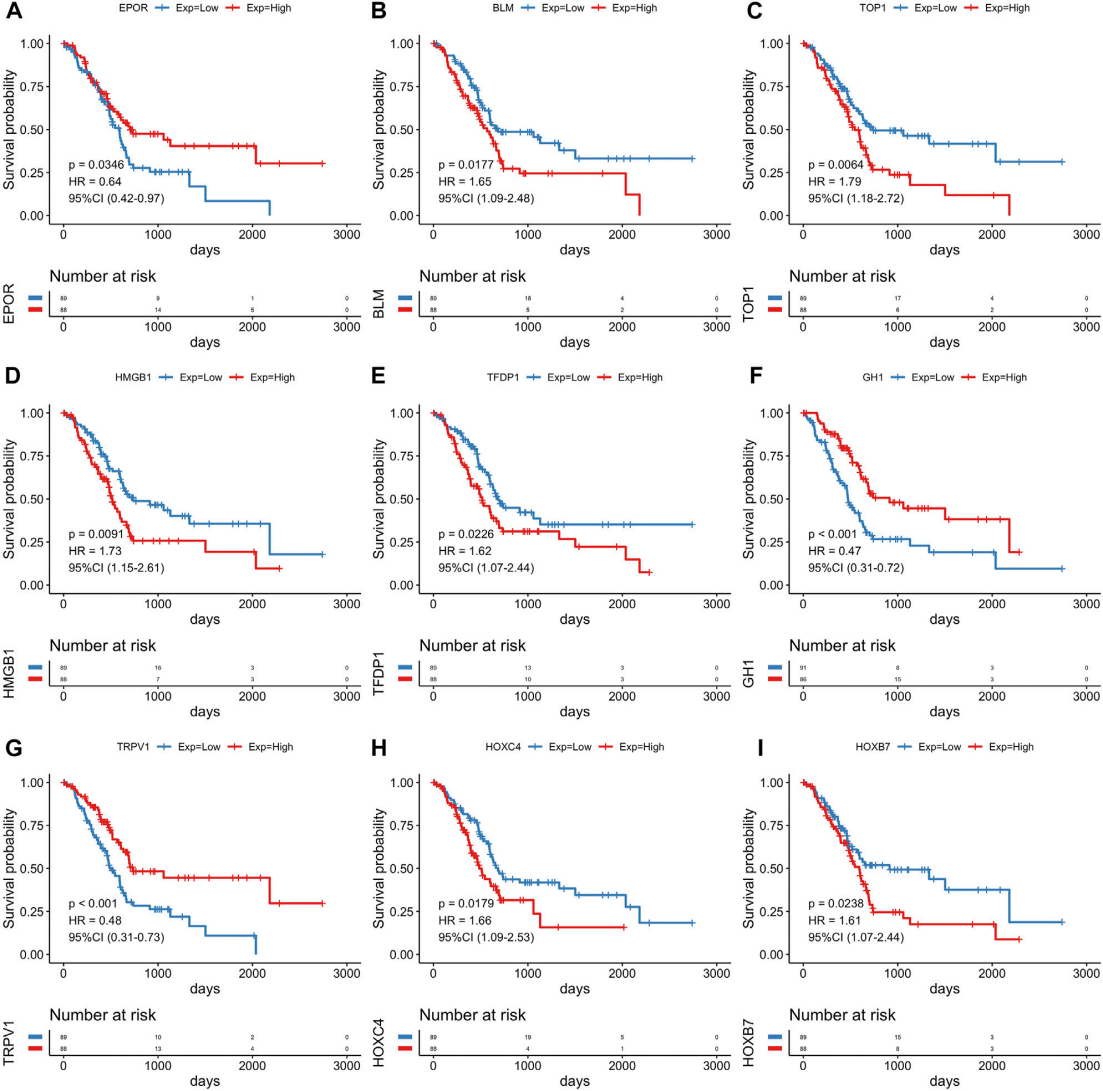
The Kaplan-Meier survival analysis of survival-related genes. Survival analysis of nine genes for PCa patients in the TCGA cohort, including EPOR**(A)**, BLM **(B)**, TOP1 **(C)**, HMGB1 **(D)**, TFDP1 **(E)**, GH1 **(F)**, TRPV1 **(G)**, HOXC4 **(H)**, HOXB7 **(I)**.

The R package limma was utilized to identify 9,020 DEGs between tumor samples and normal samples. Volcano and heatmap plot were used to visualize the DEGs (Figures 2A, B). Among these, 7,815 DEGs were upregulated and 1,205 DEGs were downregulated. Then, 179 differentially expressed aging-related genes (DE-ARGs) were selected by taking the intersection of DEGs and 307 ARGs (Figure 2C). Then, based on the OS information of 177 PCa patients from the TCGA, a univariate Cox regression analysis was performed on all the 179 DE-ARGs to predict the prognosis of PCa patients. 45 survival-related ARGs were selected and depicted in the forest plot, which contained 11 protective genes and 34 risky genes (Figure 2D). The survival curves for the survival-related genes are shown in Figure 3.

### 3.3 Construction and validation of an aging-related risk signature

**FIGURE 4:**
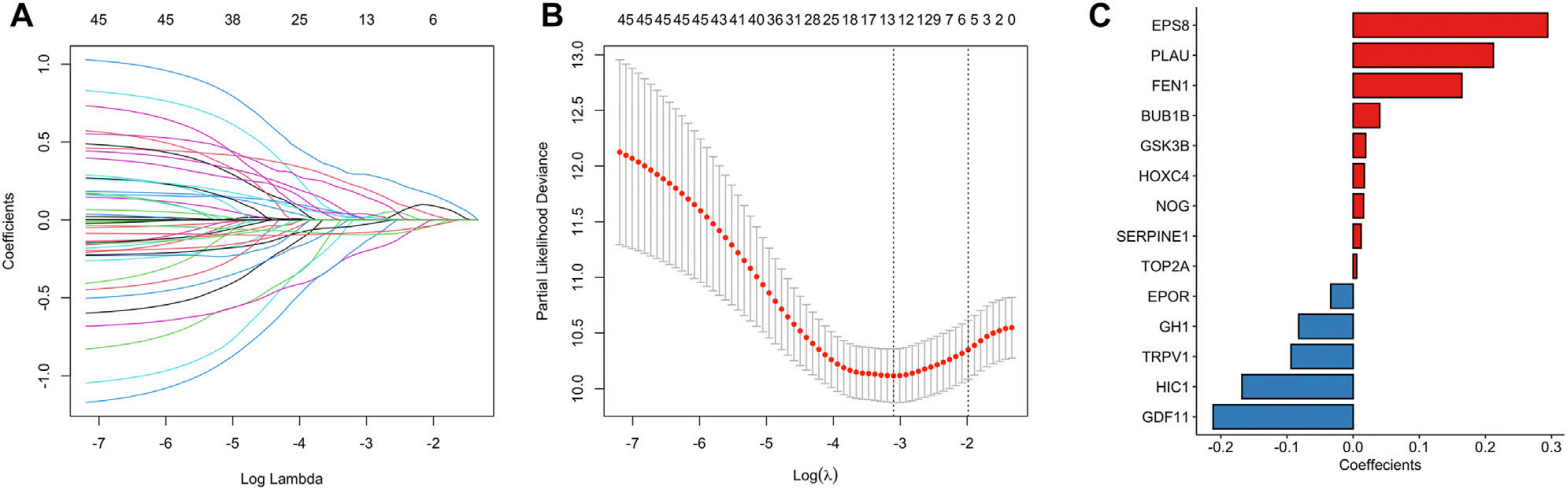
A 14 aging-related genes signature established by LASSO regression analysis. **(A)** Processes of LASSO Cox model fitting. Each curve represents an aging-related gene. **(B)** The tuning parameter (λ) was selected using 10-fold cross-validation. **(C)** LASSO coefficients of 14 key prognostic aging-related genes.

**FIGURE 5:**
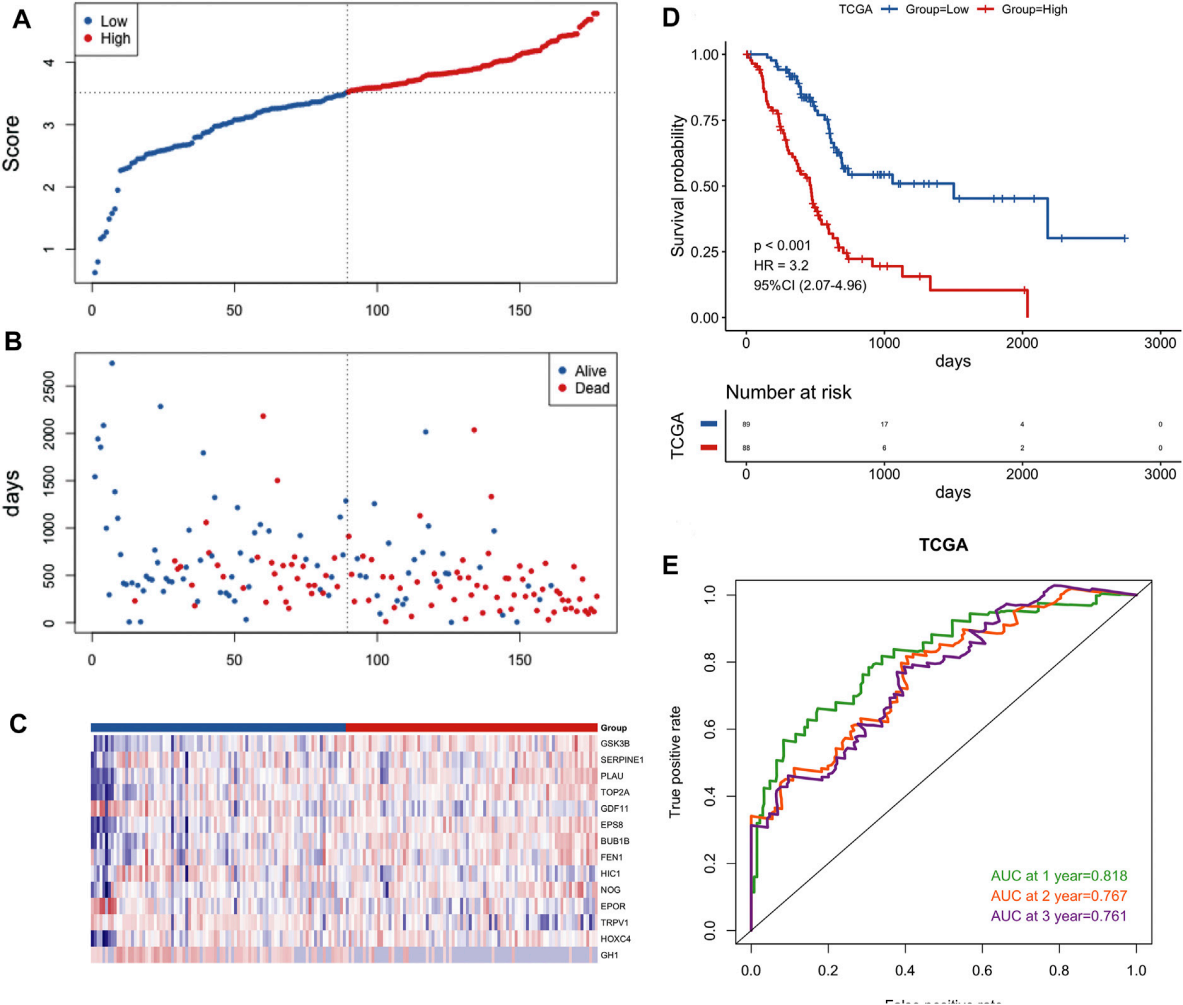
Construction of the gene signature in the TCGA cohort. **(A)** Distribution of groups based on the aging-related risk score. **(B)**The risk scatter plot of PCa patients between high- and low-risk groups. **(C)** Heatmap showed differential expression of included 14 hub genes in both groups. **(D)** Kaplan-Meier curves of overall survival of the high- and low-risk groups. **(E)** AUC prediction of 1, 2, 3-years survival rate of PCa patients.

45 prognostic survival-related ARGs were subjected to Lasso Cox analysis and 14 key prognostic ARGs were filtered out (Figure 4). Next, we built an aging-related risk signature of pancreatic cancer patients, and the risk score formula was as follows: risk score = 0.01893*GSK3B + 0.01203 * SERPINE1 + 0.21210 *PLAU +0.00538* TOP2A-0.21165 *GDF11 + 0.29423 * EPS8 + 0.04018 * BUB1B + 0.16445 * FEN1-0.16805* HIC1 + 0.01571 * NOG-0.03386 * EPOR-0.09390 * TRPV1 + 0.01691* HOXC4-0.08234* GH1. Then, we divided all PCa patients into high-risk group and low-risk group according to the median value of the risk score in the TCGA dataset (Figure 5A). Patients in the high-risk groups had higher mortality than that in the low-risk groups (Figure 5B). The gene expression heatmap analysis showed that 14 prognosticARGs were differentially expressed between the two groups (Figure 5C). The high-risk group had a lower survival rate compared to the low-risk group (Figure 5D). The AUC values of 1-, 2-, and 3-years were 0.818, 0.767, and 0.761, respectively (Figure 5E), which indicates that the model has high accuracy and stability.

**FIGURE 6:**
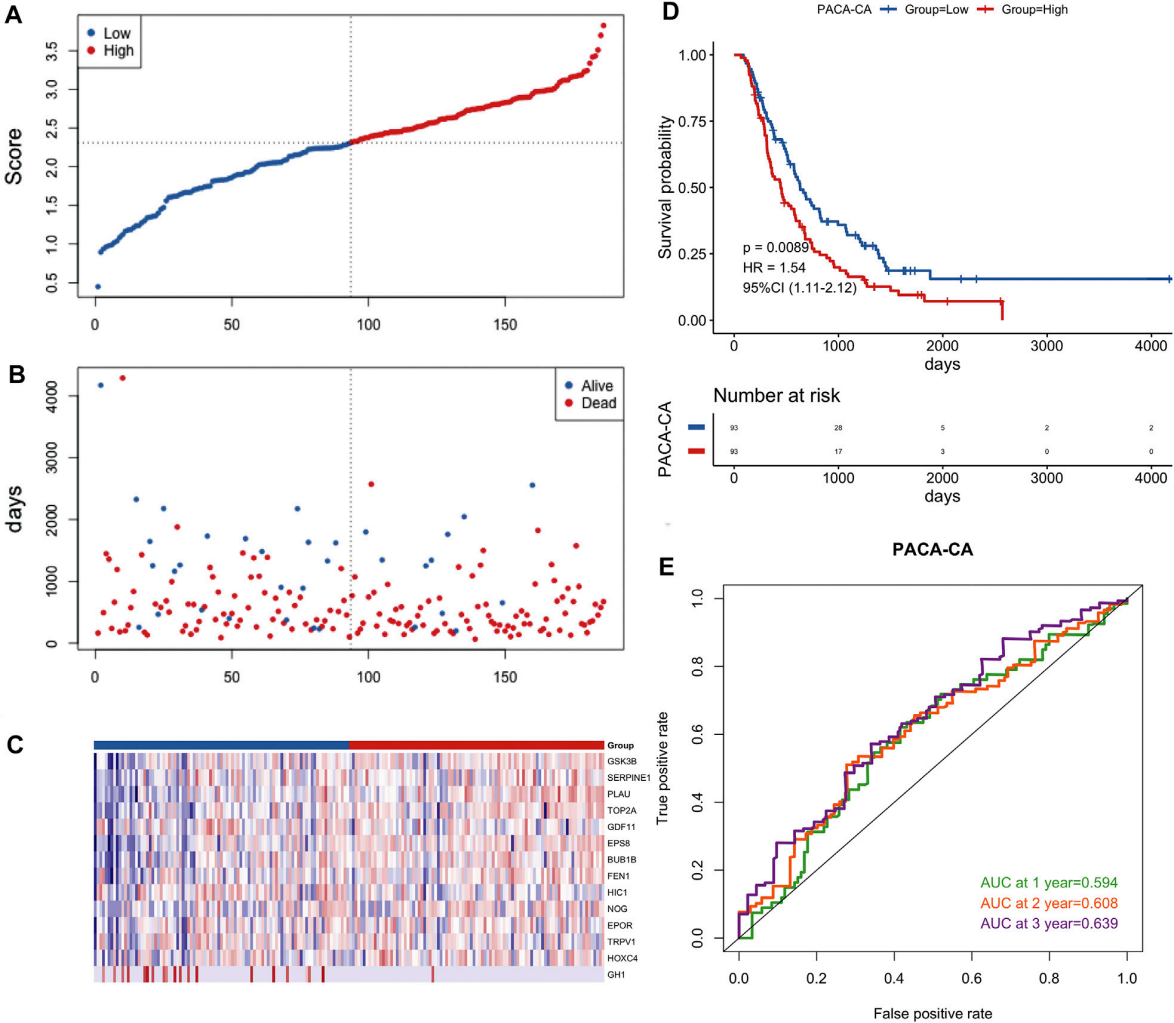
Prognostic validation of the 14-gene signature in the PACA-CA cohort. **(A)** Distribution of groups based on the aging-related risk score. **(B)**The risk scatter plot of PCa patients between high- and low-risk groups. **(C)** Heatmap showed differential expression of included 14 hub genes in both groups. **(D)** Kaplan-Meier curves of overall survival of the high- and low-risk groups. **(E)** AUC prediction of 1, 2, 3-years survival rate of PCa patients.

To further verify the validity of this signature, we performed external validation using two independent datasets, PACA-CA dataset (Figure 6) and GSE62452 dataset (Supplementary Figure S2). Similarly, the low-risk group was superior to the high-risk group in the overall survival. The mortality rate of PCa patients of the high-risk group was also higher than the low-risk group, and key prognostic ARGs found to be differentially expressed in two groups as well. The value of AUC was > 0.5 in the verification datasets, which indicated that the model also had predictive capability in other dataset.

### 3.4 Clinical relevance of risk signature

**FIGURE 7:**
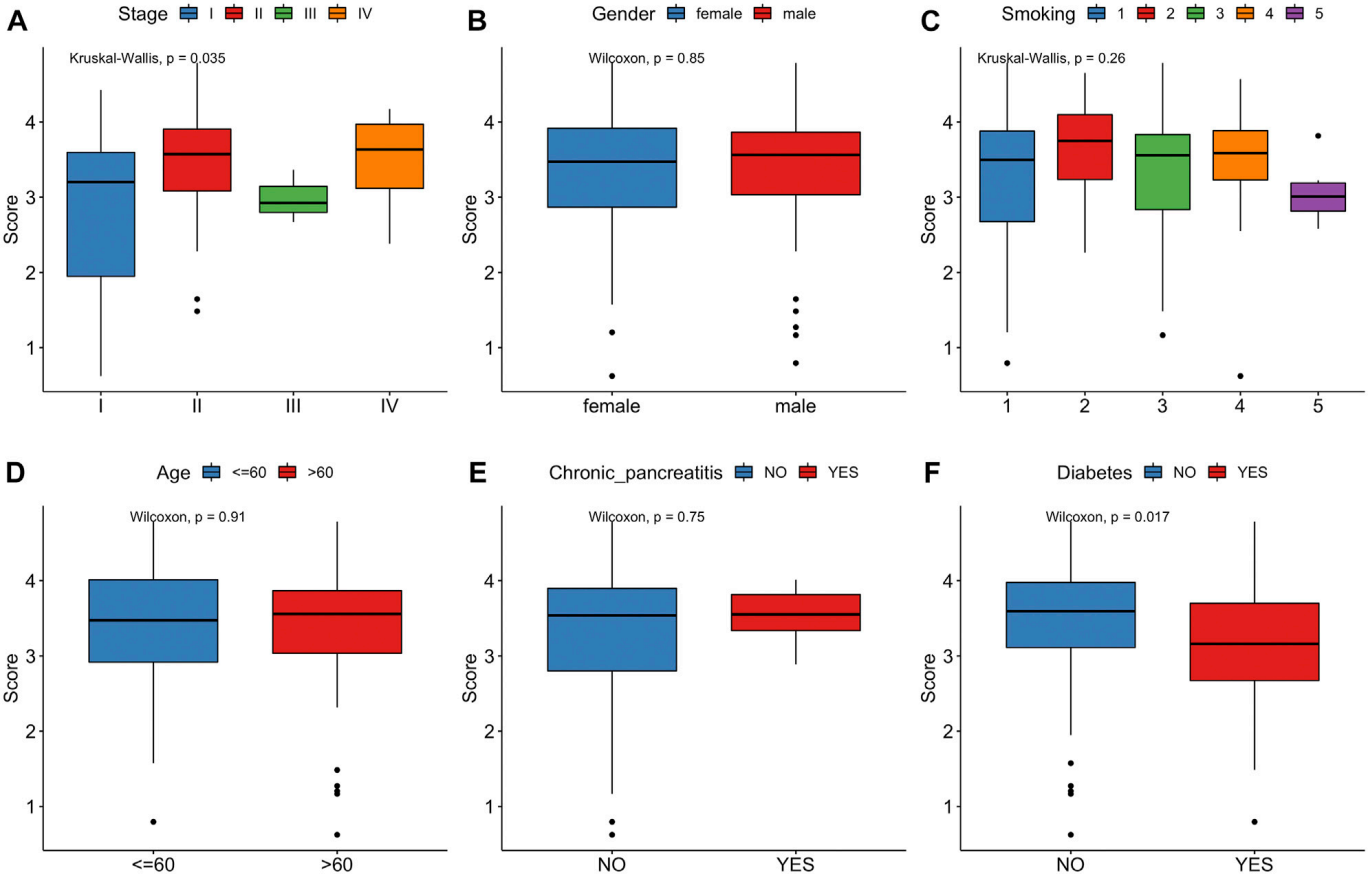
Correlation between risk score and clinical characteristics. **(A)** Stage. **(B)** Gender. **(C)** Smoking. **(D)** Age. **(E)**Chronic pancreatitis. **(F)**Diabetes.

In the TCGA cohort, we analyzed the correlation between the risk score and clinical characteristics, including stage, gender, smoking, age, chronic pancreatitis and diabetes (Figure 7). Risk score significantly increased for advanced stageof PCa. Notably, pancreatic cancer patients with a history of diabetes had a lower risk score than without a history ofdiabetes. No significant differences in age, gender, smoking status were observed between controls and PCa patients.

**FIGURE 8:**
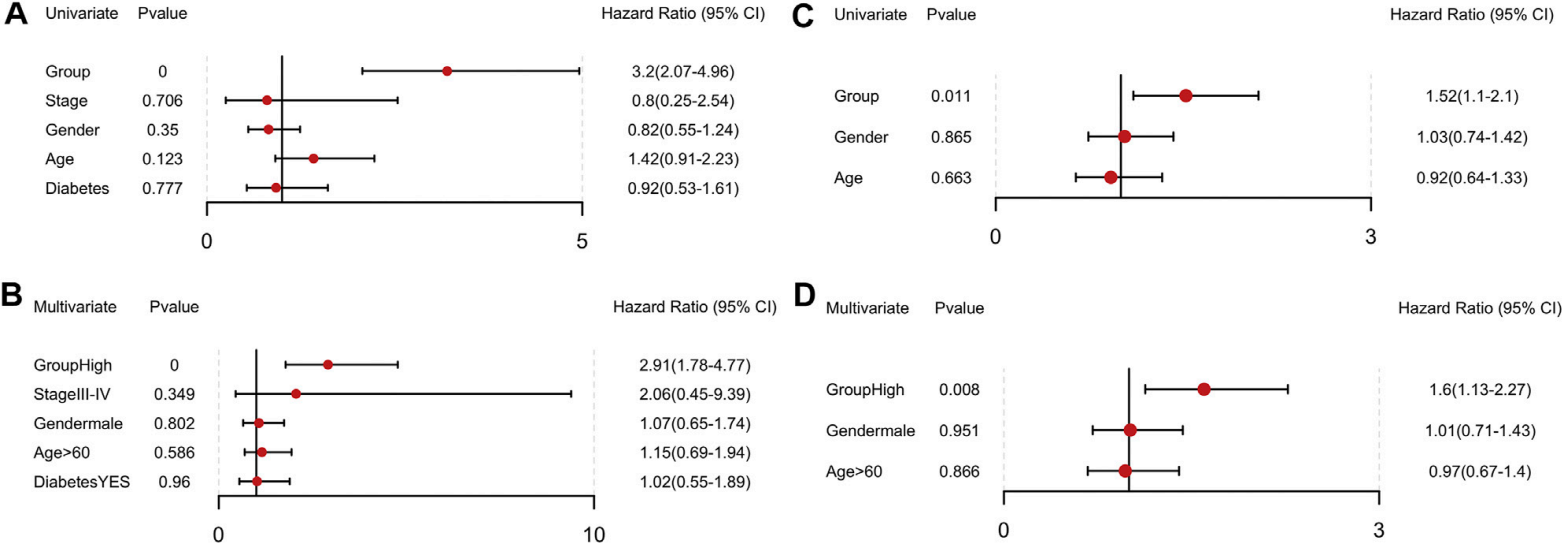
Independent prognostic value of the 14-gene signature in the TCGA and PACA-CA cohorts. **(A, B)** Univariate and multivariate COX regression analysis in the TCGA group. **(C, D)** Univariate and multivariate COX regression analysis in the PACA-CA group.

We then conducted univariate and multivariate Cox regression analyses and identified this model as an independent influencing factor for OS in the training cohort (Figures 8A, B). Verification was performed on additional independent groups PACA-CA and GSE62452 and the same conclusion was drawn (Figures 8C, D; Supplementary Figure S3). Results above indicated that our risk signature could be used for predicting the survival of PCa patients.

**FIGURE 9:**
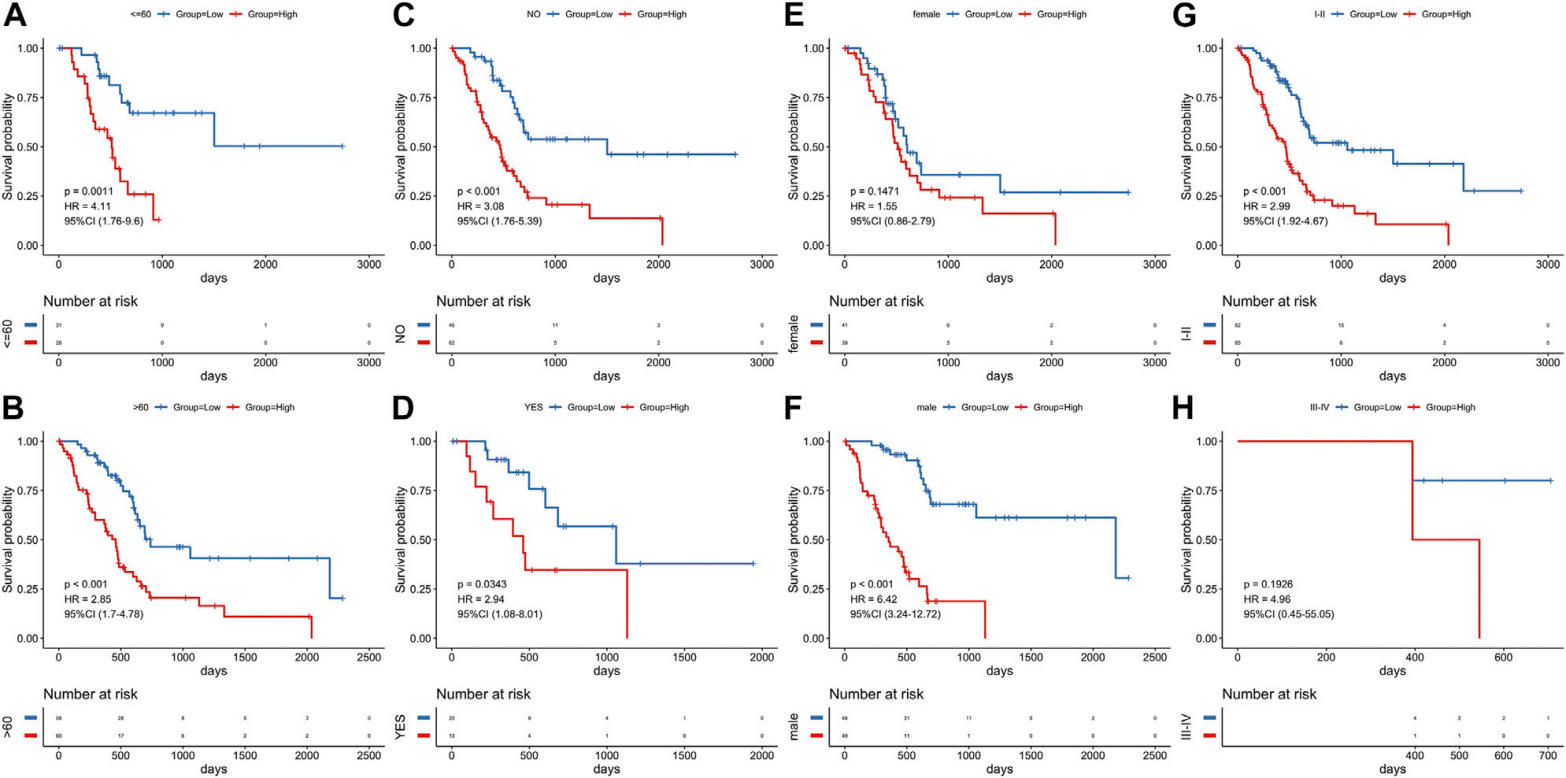
Stratified survival analysis adjusted to age, diabetes, gender and stage. All PCa patients in the training group were summarized in the stratified survival analysis, including age **(A, B)**, diabetes **(C, D)**, gender **(E, F)** and stage **(G, H)**.

In order to verify the reliability of this model, we conducted stratified analyses in the various subgroups, such as age, gender, stage, and diabetes status. We found that the risk score model had significant difference in prognosis between groups of different age groups (Figures 9A, B) and groups with different diabetes history (Figures 9C, D). There were significant prognostic differences in male groups and in stage I-II (Figures 9F, G). Although there is no significant difference in prognosis among other groups, the trend is the same (Figures 9E, H), indicating that the model had a perfect predictive ability for evaluating prognosis.

### 3.5 Building and evaluation of a prognostic nomogram

**FIGURE 10:**
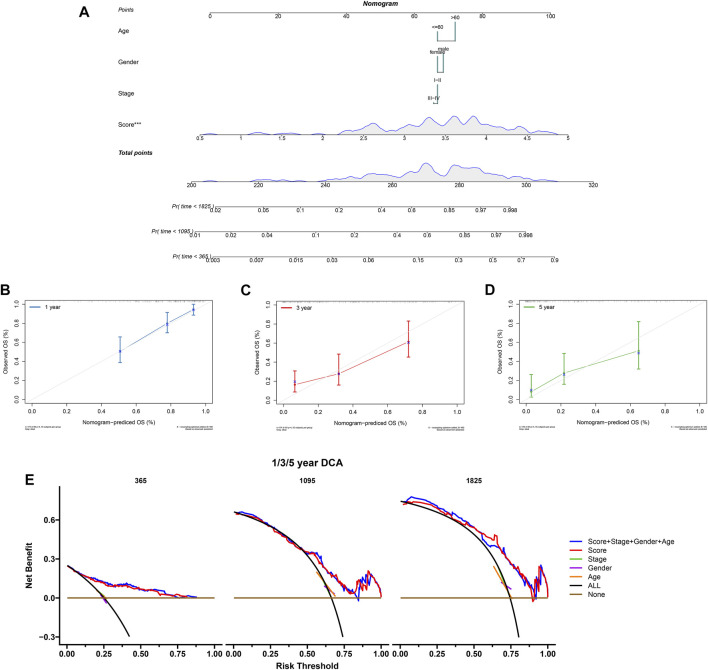
Construction and validation of the nomogram. **(A)** The nomogram combing risk score and clinical factors. **(B–D)** The calibration curve of the nomogram for predicting the 1-, 3-, and 5-years survival of PCa patients. **(E)** The DCA curves showed the expected net benefits of the nomogram prediction for 1-, 3-, and 5-years overall survival. None: represented an event will occur in no patients have 1-, 3- or 5-years survival; All: represented an event will occur in all patients have 1-, 3- or 5-years survival under a certain threshold rate.

To predict PCa patients’ survival probability, a nomogram was developed based on risk score, stage, gender, and age (Figure 10A). As shown in the nomogram, risk score had the greatest impact on the survival outcome of patients. The calibration curve indicated the high performance of the nomogram in predicting OS (Figures 10B–D). The DCA analysis also demonstrated that our combined prognostic model showed the best net benefit for 1-year, 3-years, and 5-years OS (Figure 10E). These results indicate that our nomogram has a good predictive ability in the survival probability of PCa patients.

### 3.6 Correlations with immune microenvironment

**FIGURE 11:**
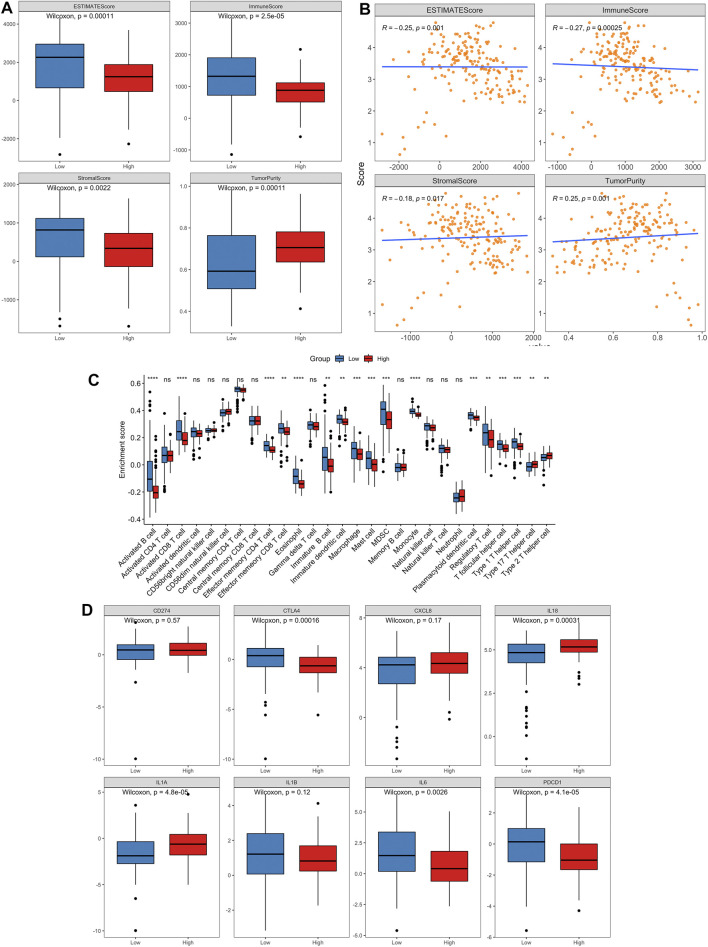
The landscape of immune infiltration in high- and low-risk PCa patients.**(A)** Comparisons of ESTIMATE score, immune score, stromal score and tumor purity between the high- and low-risk groups. **(B)** Pearson correlation analysis between the risk score and ESTIMATE score, immune score, stromal score and tumor purity, respectively. **(C)** Comparison of tumor-infiltrating immune-cell fraction between the high- and low-risk groups using ssGSEA method. **(D)** Box plots visualizing significantly different immune checkpoints and pro-inflammatory factors between high-risk and low-risk patients.

Aging is associated with immune remodeling, including alterations in T-cell-mediated immune function and immune dysregulation. Based on TCGA training set, the “ESTIMATE” R package was used to calculate the immune score, stromal score, ESTIMATE score and tumor purity of every cancer sample. The distribution of four indexes in high risk and low risk groups was compared. The results demonstrated that the immune-, stromal- and ESTIMATE-score in low risk group were noticeably higher than those in high risk group, and the tumor purity was statistically lower than that in high-risk group (Figure 11A). At the same time, the Spearman correlation between these four indicators and risk score was calculated. The risk score had a negative relation with immune score, stromal score and ESTIMATE score and positive correlation with tumor purity (Figure 11B).

To further explore whether our aging-related risk model reflected the immune state of the TME in PCa, we used CIBERSORT, ssGSEA and xCELL to calculate the infiltration scores of different immune cells and analyze the differences between high-risk and low-risk groups. The results of ssGSEA method revealed that the low-risk group has a relatively higher level of immune cell (activated B cell, activated CD8 T cell and macrophage) infiltrations than the high-risk group (Figure 11C). Similar results are also obtained for the other two methods (Supplementary Figures S4, S5).

On the other hand, we compared the expression differences of important immune checkpoints and proinflammatory factors between two risk groups. The levels of CTLA4, IL6 and PDCD1 expression in low-risk group were markedly greater than those in high-risk group, and the levels of IL18 and IL1A expression were significantly lower than those in high-risk group (*p* < 0.05, Figure 11D).

### 3.7 Screening therapeutic drugs for high-risk PCa patients

**FIGURE 12:**
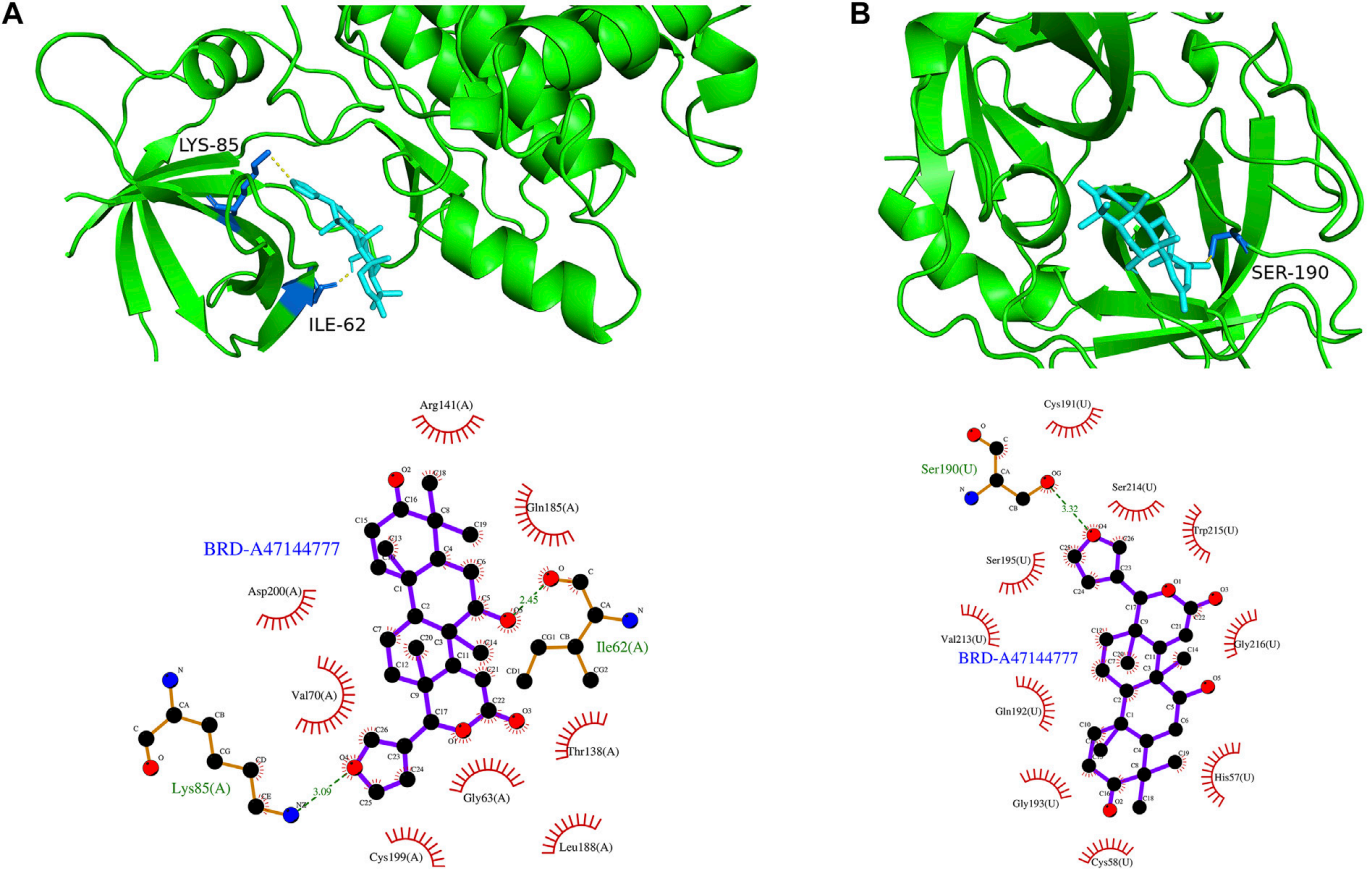
The result of molecular docking between candidate drugs and coregenes. **(A)** The molecular docking results between BRD-A47144777 and GSK3B. **(B)** The molecular docking results between BRD-A47144777 and PLAU.

The top 200 DEGs (100 up-regulated DEGs and 100 down-regulated DEGs) in the high-risk group were selected and entered as the inputs of the Cmap database. The top 10 molecules with positive connectivity value were selected as potential drugs. Among them, five molecules (BRD-A47144777, BRD-A58955223, BRD-K48300629, BRD-K68132782 and BRD-K73109821) have structural data. The protein structure information of high-risk group core genes (PLAU, GSK3B) was downloaded from the PDB database. To verify whether the five predicted drugs interact with the core genes, molecular docking technology was performed. As shown in Table 2, the docking affinity between all five predicted drugs and core genes is less than–5 kcal/mol. It indicates that there is a stable binding force between the predicted drugs and the core genes. Especially, the small molecule BRD-A47144777 has the excellent binding ability with GSK3B (−8.2 kcal/mol) and PLAU (−8.8 kcal/mol) (Figure 12). BRD-A47144777 binds to GSK3B through interacting with amino acid residues, such aslys85, ile62, arg141, asp200, val70, gln185, gly63, thr138, cys199 and leu188. BRD-A47144777 binds to PLAU by interacting with amino acid residues, such as ser190, ser195, val213, gln192, gly193, cys58, cys191, ser214, trp215, gly216 and his57. These results indicate that BRD-A47144777 has a good combination with its target and has the potential to become a therapeutic drug for PCa.

**TABLE 2:** Docking affinity score between core genes and the predicted drugs.

Core genes	Predicted drugs	Affinityscore (kcal/mol)
GSK3B	BRD-A47144777	−8.2
	BRD-A58955223	−7.1
	BRD-K48300629	−6.4
	BRD-K68132782	−8.5
	BRD-K73109821	−7.1
PLAU	BRD-A47144777	−8.8
	BRD-A58955223	−6.7
	BRD-K48300629	−5.9
	BRD-K68132782	−5.4
	BRD-K73109821	−6.7

### 3.8 Protein expression level of ARGs and survival analysis

**FIGURE 13:**
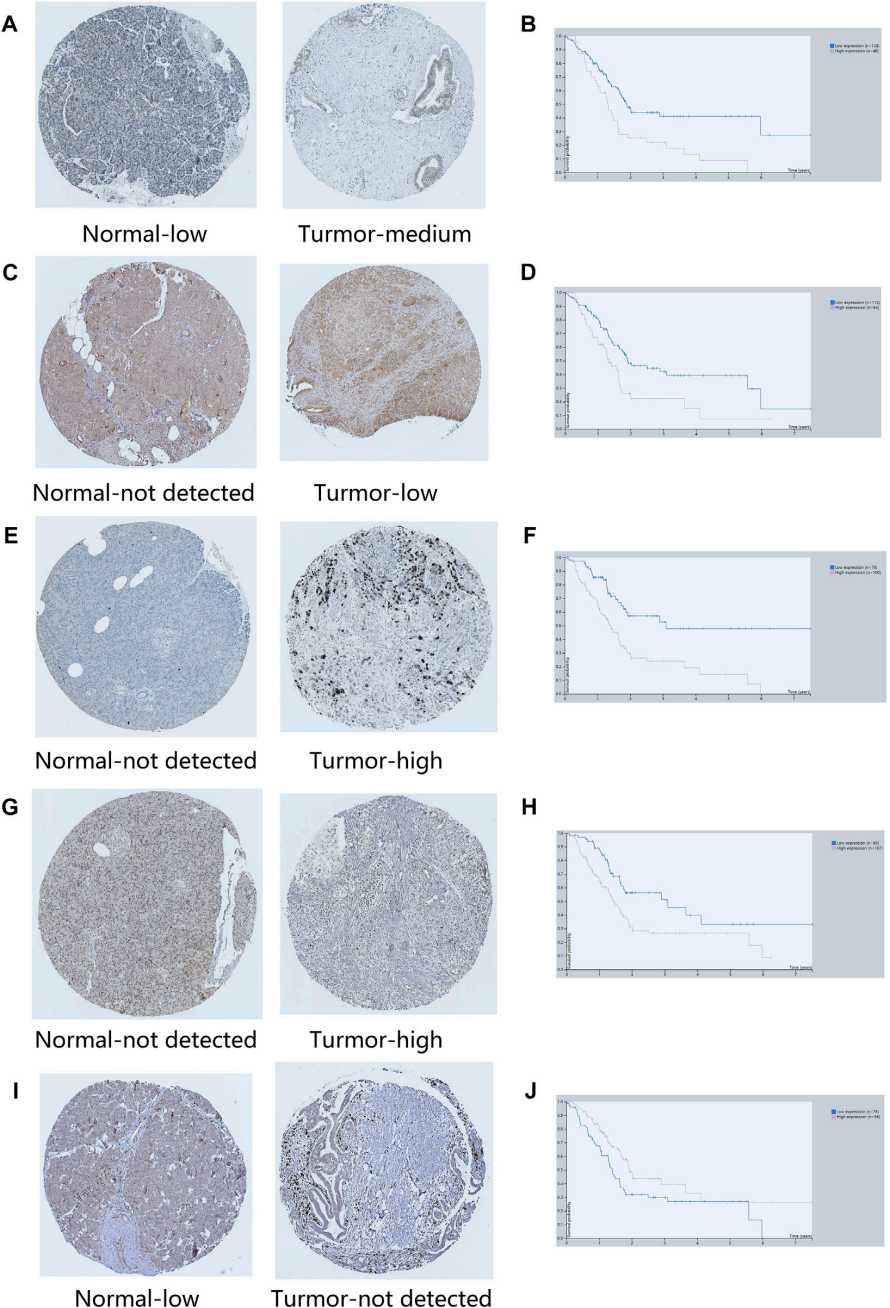
Immunohistochemical results of key ARGs expressions and survival analysis. **(A–B)** GSK3B. **(C–D)** SERPINE1. **(E–F)** TOP2A. **(G–H)** FEN1. **(I–J)** HIC1.

In order to verify the expression of ARGs in normal and tumor tissues, we obtained immunohistochemical results and survival results from HPA database. Except BUB1B, EPOR, TRPV1 and HOXC4, all ARGs had immunohistochemical results. The protein expression levels of GSK3B, SERPINE1, TOP2A, FEN1 and HIC1 were consistent with the signature (Figure 13). Although the protein expressions of PLAU, GDF11, EPS8 and GH1 in tumor and normal groups were not significantly different, the survival analysis results were consistent with our risk model results. It is noteworthy that NOG was lower expressed in tumor tissues than normal tissues in HPA database, which is contrary to its survival results.

## 4 Discussion

PCa is a lethal disease which divided into adenocarcinoma and rare endocrine tumors (Han and Von Hoff, 2013). Because the pancreas lies deep in the abdomen, PCa is difficult to detect and dignose (Moore and Donahue, 2019). Metastasis is also an important feature in PCa progression (Zhou et al., 2018). Despite new therapy strategies and methods have been developed, the5-year survival rate of PCa has changed little over the past several decades (Karapanos and Nomikos, 2011; Neoptolemos et al., 2018). It is estimated that PCa will become the second leading cause of cancer related mortality in western countries in the next ten years (Neoptolemos et al., 2018). Hence, it is quite necessary to develop novel therapeutic targets and prognostic indicators to improve the outcomes of patients with PCa.

There are six hallmarks in the development and progression of cancers, including maintaining proliferative status, evading growth suppressors, resisting cell death, enabling replicative immortality, inducing angiogenesis, and activating invasion and metastasis. The basis of these six hallmarks is genomic instability (Hanahan and Weinberg, 2011). As with cancer, genomic instability is also one of the important hallmarks of aging (Lopez-Otin et al., 2013). Cancer and aging can be seen as two different manifestations of the same process, namely cell damage accumulation. In addition, aging is an effective barrier to prevent cancer (Calcinotto et al., 2019). Consequently, establishing aging-related risk model and inducing cancer cell senescence may also become a new therapeutic strategy for pancreatic cancer.

Because public databases and bioinformatics tools are available, more prognostic risk models are used to assess the OS of PCa patients. Nevertheless, among the numerous risk models, the aging-related prognostic model of PCa has never been reported.

In this study, we firstly perform GSEA enrichment analysis of coding genes of tumor samples and normal samples in 80 aging-related gene sets, the result showed that tumor samples were significantly enriched in GOBP_ AGING, RODWELL_ AGING_ KIDNEY_ UP, LY_ AGING_ MIDDLE_ Up, Lee_ AGING_ CEREBELLUM_ Up, and other senescence-associated molecular signal pathways. These findings demonstrated that aging process was involved in the progression of PCa. Previous studies have shown that aging is a risk for PCa (Matsuda et al., 2019; Cheng et al., 2020).

To find out which genes are associated with the aging process in PCa, we used DEG analysis. We identified 9,020 DEGs and 179 differentially expressed aging-related genes. Based on survival information of PCa patients, we screened 45 prognostic survival-related genes, 34 risky genes and 11 protective genes were included. Furthermore, LASSO regression analyses identified 14 key prognostic genes (GSK3B, SERPINE1, PLAU, TOP2A, GDF11, EPS8, BUB1B, FEN1, HIC1, NOG, EPOR, TRPV1, HOXC4, GH1), which were used to build the prognostic risk model. We found that these hub genes clustered into three main groups including cancer-promoting factors, tumor suppressors, and biomarkers. Class I genes played an essential role in pancreatic carcinogenesis, consisting of GSK3B, PLAU, TOP2A, BUB1B, SERPINE1, EPS8. Glycogen synthase kinase-3 β (GSK3B) is a widely expressed serine/threonine kinase that plays an important role in vital cellular processes including cell proliferation, DNA repair, cell cycle progression, signal transduction and metabolic pathways (Pecoraro et al., 2021). Inhibiting its expression can kill PCa cells and slow down the growth and metastasis of PCa (Edderkaoui et al., 2018). Therapeutic agents targeting GSK3β that could surmount chemoresistance of PCa (Pecoraro et al., 2021). Similarly, suppressing GSK3β could delay kidney aging (Fang et al., 2022). Expression and secretion of PLAU in senescent cells mediates cell proliferation and apoptosis (Hildenbrand et al., 2008). PLAU activated endothelial mesenchymal transformation (EMT) progression in pancreatic cancer (Zhao et al., 2020). TOP2A activates β-catenin pathway and EMT process in pancreatic cancer (Pei et al., 2018). Down-regulating PLAU or TOP2A can inhibit pancreatic cancer cell proliferation and migration (Pei et al., 2018; Zhao et al., 2020). BUB1B is the hub gene of pancreatic ductal adenocarcinoma (PDAC), and the expression of BUBR1 encoded by BUB1B can predict poor prognosis in pancreatobiliary-type tumors (Gladhaug et al., 2010; Dong et al., 2019). The upregulation of BUB1B in tumor tissues are related to worse OS and disease-free survival (DFS) in PDAC (Dong et al., 2019). SERPINE1 serve as a miR-34a target, which is negatively-related to the survival of PDAC patients. The protein plasminogen activator inhibitor (PAI-1) encoded by SERPINE1 mediates the proliferation and invasion of PDAC-derived cell lines (Akula et al., 2020). Eps8 is significantly increased in pancreatic cancer, and its expression is positively correlated with the migration potential of tumor cells (Welsch et al., 2007; Ohshima et al., 2019). GDF11 is a tumor suppressor, Class Ⅱ genes are mainly tumor suppressor, including GDF11, TRPV1 and HIC1. GDF11 was remarkably downregulated in PCa, which inhibited tumor growth by promoting apoptosis of pancreatic cancer cells. Overexpression of GDF11 can inhibit the proliferation, migration and invasion *in vitro* (Liu et al., 2018). TRPV1 is involved in sensing cancer pain, and is a potential target for inhibiting of cancer pain in pancreatic cancer (Prevarskaya et al., 2007). TRPV1 overexpression inhibits cell proliferation (Huang et al., 2020a). HIC1 is downregulated in PCa and act as a STAT3 inhibitor, which may be a promising target of cancer research and treatment for PCa (Hu et al., 2016). The role of other genes in the development of pancreatic cancer is not fully clarified, and they often participate in the cancer pathway as a part. Most tertiary lymphoid structures did not have any FEN1 expression, but when present, it is an independent predictor of decreased DFS and worse recurrence-free survival (RFS) of PCa with higher efficiency than that of TN classification (Isohookana et al., 2018). Noggin (NOG) is a component of the TGF-β signaling system in PCa (Kokaji et al., 2018). EpoR participates in PI3K/Akt signal transduction in PDAC cells under the mediation of erythropoietin (Welsch et al., 2011). The expression level of HOXC4 mRNA in tumor group was significantly higher than that in normal samples. HOXC4 is the target of miR-455-3p and its formation of miR-455-3p-HOXC4 axis may be closely related to the metastasis and prognosis of human pancreatic cancer (Shang et al., 2021). GH1 participates in the formation of GIPC1, which is necessary for integrin circulation during cell migration, angiogenesis and cytokinesis. Upregulation of GIPC1 in pancreatic cancer promotes tumor proliferation and invasion (Katoh, 2013). The protein expression level and survival results of GSK3B, SERPINE1, TOP2A, FEN1 and HIC1 confirmed the reliability of our risk model.

The risk factors of PCa contained family history, obesity, type 2 diabetes and tobacco use (Mizrahi et al., 2020). Our aging-related signature could be used as an independent prognostic factor. In this study, we analyzed the correlation between the aging-related risk score and clinical features. Our result showed that the risk score was positively associated with advanced tumor stage. Lei Huang et al. (Huang et al., 2018) counted the 3-years survival rates of 125,183 PCa patients at different stages stratified by age, and the results showed that 1) in the early stages (I-II), the survival rate decreased sharply with age; 2) in the population over 70 years old, the survival rate of patients in late stage (III-IV) decreased sharply compared with that in early stages (I-II). Furthermore, pancreatic cancer patients with a history of diabetes had a lower aging-related risk score than without a history of diabetes in our study. Cell senescence is considered as the main mechanism for the development and progression of type 2 diabetes mellitus, and diabetes mellitus is a main risk factor of the cancer (Burton and Faragher, 2018). At the same time, in some epidemiological studies, the risk of pancreatic cancer is significantly higher in new-onset diabetic patients compared with long-standing diabetes (Pizzato et al., 2019). Maybe this is the reason why PCa patients have lower aging-related risk score with a history of diabetes than without a history of diabetes. Of course, more clinical information is needed to verify this result in the future. For clinical convenience, we integrated risk scores and several clinical variables to establish a nomogram. The calibration curve and DCA showed that the nomogram combined with 14-gene signature and conventional clinical prognostic factors was excellent in predicting the survival rate (1-year, 3-years and 5-years) of patients with PCa. Taken together, we believed that the aging-related gene signature in our study is a useful indicator for PCa survival that could be applied to clinical practice.

A key factor in the lethality of PCa is acquired immunity privilege, which is driven by immuno-suppressive tumor microenvironment, poor infiltration of T cells and low mutation burden (Morrison et al., 2018). The immune score was positively correlated with the survival rate and good prognosis of the patients (Huang et al., 2020b; Bi et al., 2020). We compared the relationship between risk score and immune microenvironment. Our results demonstrate that the low-risk group had lower tumor purity and higher immune score, stromal score and ESTIMATE score compared to high-risk group. Spearman correlation analysis showed that the aging-related risk score was significantly negatively correlated with immune score, stromal score and ESTIMATE score, and positively correlated with tumor purity. These results suggested that there was a correlation between risk score and immune microenvironment. Besides, this study indicated that the infiltration scores of immune cell (activated B cell, activated CD8 T cell and macrophage) in low-risk group were significantly greater than those in high-risk group. Immunotherapy based on immune checkpoint blockade represents a promising modality in pancreatic cancer (Kunk et al., 2016). Thus, we analyzed the correlationship between immune checkpoints and aging-related risk score in the training cohort. Results showed that the expression of immunosuppression-related genes (CTLA4, IL6and PDCD1) had a negative correlation with aging-related risk score. Immune checkpoints by anti-CTLA-4 and/or anti-PD-1/anti-PD-L1 agents have been developed in PCa (Schizas et al., 2020). Hence, inducing cell senescence may play an antitumor effect through immunosuppression. Furthermore, results of our study showed that, increased levels of proinflammatory factors such as cytokines (IL1A and IL18) in high-risk group vs. low-risk group. IL1A/IL-1R1 signaling was involved in pancreatic cancer cell migration (Tjomsland et al., 2016). IL-18 is a negative prognostic marker for survival and of PCa patients (Li et al., 2019).

In order to further exert the efficacy of this aging-gene signature, we identified BRD-A47144777 as a potential therapeutic drug through the Cmap database. BRD-A47144777 (dihydro-7-desacetyldeoxygedunin) is a HSP90 inhibitor, and its effect has not been reported yet. HSP90 participates in heat shock response (HSR), which is responsible for stress release and refolding of denatured proteins. Cancer, aging, diabetes, cardiovascular disease and many other pathological conditions will have a negative impact on HSR function (Kurop et al., 2021). Overexpression of HSP90 is a factor in tumorigenesis, and monotherapy with HSP90 inhibitors has shown some success in treating advanced solid tumors (Shimomura et al., 2019; Birbo et al., 2021). The research also showed that Hsp90 inhibitors are senolytic drugs to extend healthy aging (Fuhrmann-Stroissnigg et al., 2018). Senolytics is a new type of agent that can delay human aging, and it is also the first therapeutic drug mainly used to prolong human life through clinical trials (Kirkland et al., 2017). Therefore, BRD-A47144777 is a promising drug to treat high-risk PCa patients. However, further experimental analysis was needed to prove it.

The study of aging-related prognostic models in cancer has attracted more and more attention. Xue et al. (2022) identified a novel seven-ARG risk signature to predict prognoses of pancreatic adenocarcinoma. Compared with it, our prognosis model has higher precision. The ARGs that overlap our prognostic model with its model are PLAU and TOP2A. In addition, PLAU has predictive value as ARG in breast cancer, lung square cancer, head and neck square cell cancer, etc (Yang et al., 2020; Lv et al., 2021; Zhai et al., 2022). TOP2A can predict the prognosis of hepatocellular carcinoma (Cai et al., 2022). It suggests that PLAU and TOP2A may be dangerous biomarkers and potential therapeutic targets for PCa and other cancers.

In this study, we used single factor cox regression analysis and LASSO regression analysis to build a prognostic risk score model, which is the most common and basic method in bioinformatics analysis, following with developing a nomogram based on risk score. The AUC value, calibration curve and DCA showed that risk score model and the nomogram had good validity and reliability. In fact, these methods have been widely used to construct prognostic risk models. Nearly 1,000 articles were published every year on the construction of prognostic risk model based on this method through PubMed database. In addition, this method is convenient and feasible. In this study, we constructed a 14 aging-related genes signature, which could provide valuable information for clinicians to calculate the prognostic risk value of any pancreatic cancer patient through a simple risk score formula. In addition, we developed a nomogram was based on risk score, stage, gender and age. The clinician can get the relevant point by the position of these data in the nomogram, and obtain the survival probability of the patient by calculating the total points of each feature. The only difficulty is gene detection and standardization. However, this study has some shortcomings. First, *in vitro* and *in vivo* experiments are needed to verify our findings. Second, we need more samples to further verify our signature.

## 5 Conclusion

In summary, this study has identified 14 hub ARGs related to prognosis and developed an aging-related risk signature for predicting PCa prognosis using the publicly available TCGA database. It was evaluated and verified in the training set and verification set. Our prognostic aging-related signature can also predict the severity of PCa and the immune cell infiltration level. This risk signature has a strong predictive performance for the prediction of PCa prognosis, and it may serve as a potential therapeutic target of PCa patients.

## Data Availability

Publicly available datasets were analyzed in this study. This data can be found here: https://xenabrowser.net/datapages/.

## Abbreviations

TCGA, The cancer genome atlas database; Cmap, Connectivity map; DCA, Decision curve analysis; HPA, Human protein atlas; PCa, Pancreatic cancer; MSigDB, The molecular signatures database; ARGs, Aging-related genes; HAGR, Human ageing genomic resources database; GSEA, Gene set enrichment analysis; DEGs, Differentially expressed genes; LASSO, Least absolute shrinkage and selection operator; OS, Overall survival; AUC, Area under the curve; ROC, The receiver operating characteristic; TME, Tumor microenvironment; ssGSEA, Single sample gene set enrichment analysis; DE-ARGs, Differentially expressed aging-related genes; DFS, Disease-free survival; EMT, Endothelial mesenchymal transformation; PDAC, Pancreatic ductal adenocarcinoma; HSR, Heat shock response.
