# Supplementary material for: Construction and validation of an aging-related gene signature predicting the prognosis of pancreatic cancer
Source: Front Genet. 2023 Jan 18;14:1022265. doi: 10.3389/fgene.2023.1022265 (PMC9889561; doi:10.3389/fgene.2023.1022265)
Supplement: Supplementary file 1 [file Table1.DOCX]

Supplementary Material

## Supplementary Figures

**
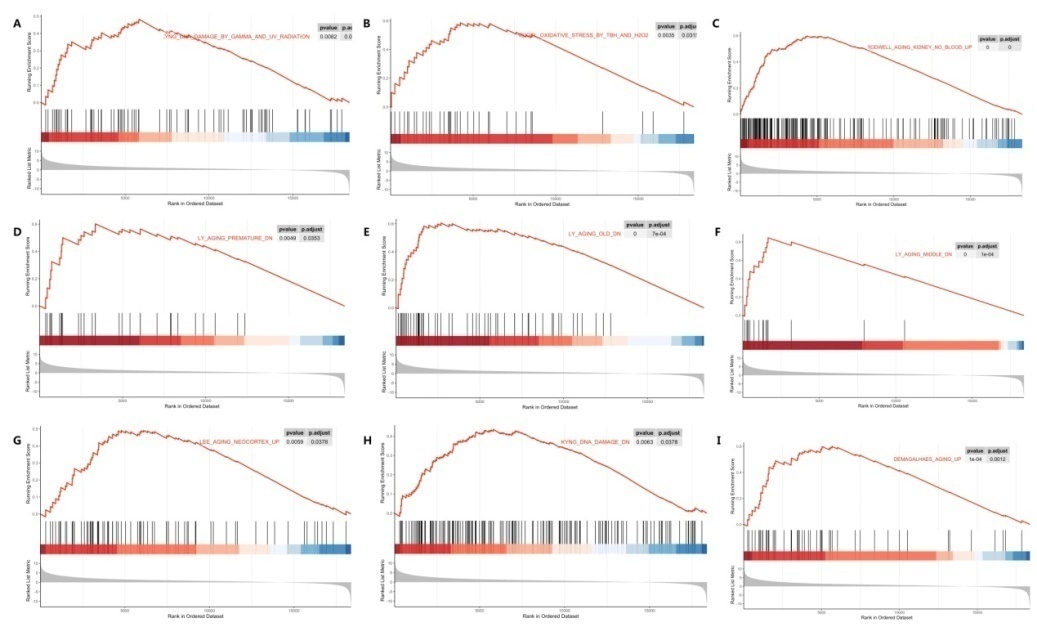
**

**Supplementary Figure S1. Gene Set Enrichment Analysis (GSEA).** GSEA analysis of the KYNG_DNA_DAMAGE_BY_GAMMA_AND_UV_RADIATION(A), WEIGEL_OXIDATIVE_STRESS_BY_TBH_AND_H2O2 (B), RODWELL_AGING_KIDNEY_NO_BLOOD_UP (C), LY_AGING_PREMATURE_DN (D), LY_AGING_OLD_DN (E), LY_AGING_MIDDLE_DN(F), LEE_AGING_NEOCORTEX_UP (G),KYNG_DNA_DAMAGE_DN (H) and DEMAGALHAES_AGING_UP (I).

**
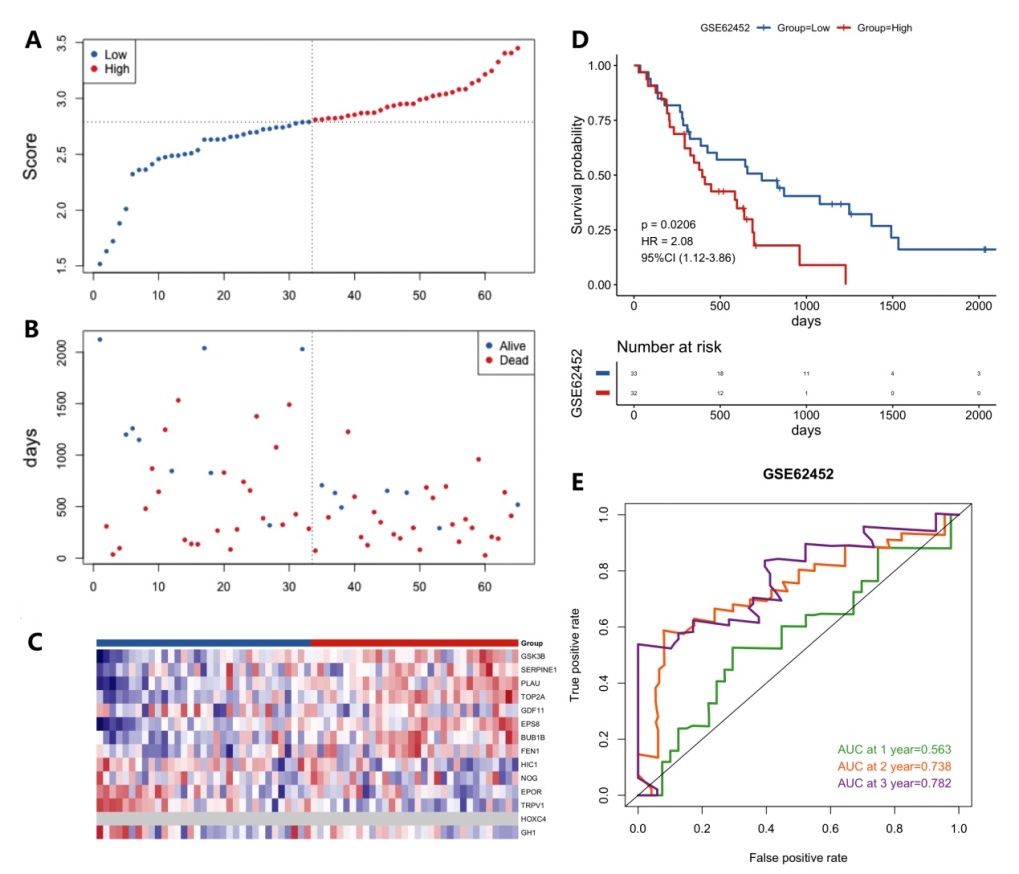
**

**Supplementary Figure S2. Prognostic validation of the 14-gene signature in the GSE62452dataset.** (A) Distribution of groups based on the aging-related risk score. (B)The risk scatter plot of PCa patients between high- and low-risk groups. (C) Heatmap showed differential expression of included 14 hub genes in both groups. (D) Kaplan-Meier curves of overall survival of the high- and low-risk groups. (E) AUC prediction of 1, 2, 3-year survival rate of PCa patients.

**
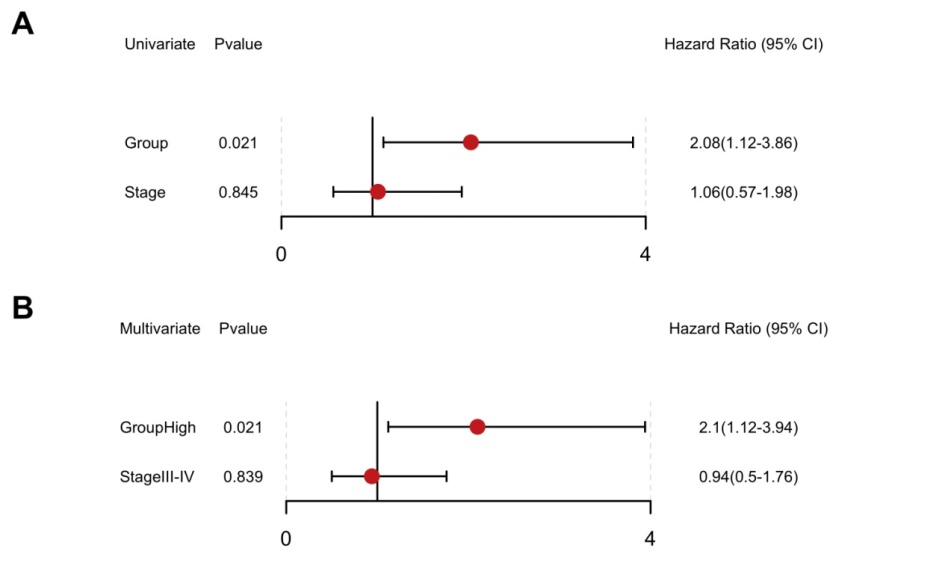
**

**Supplementary Figure S3. Independent prognostic value of the 14-gene signature in the GSE62452dataset.** Univariate (A) and multivariate(B) COX regression analysis in the GSE62452 dataset.

**
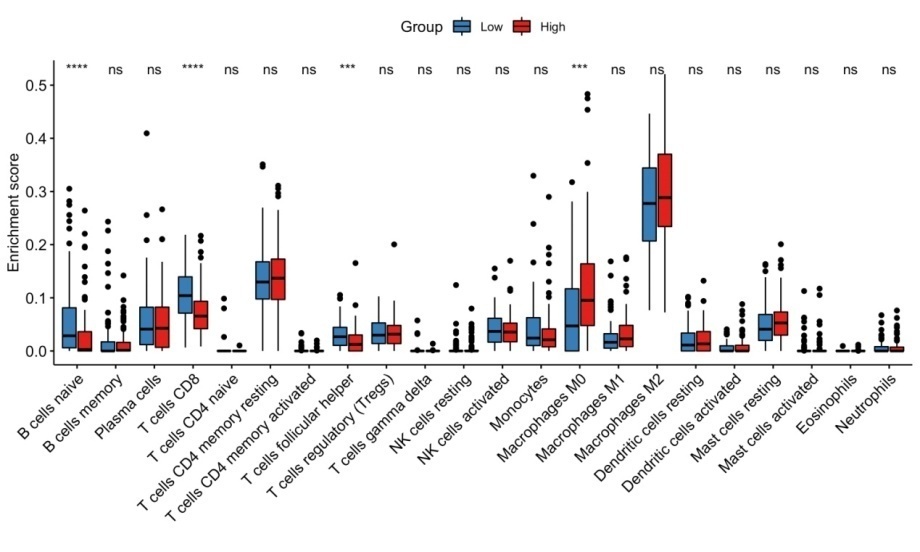
**

**Supplementary Figures S4.The comparison of immune-cell fractions between the high- and low-risk groups using CIBERSORT.**

**
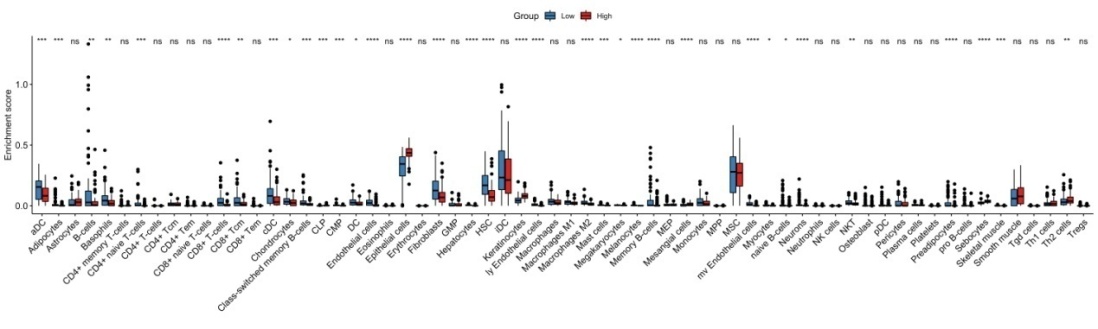
**

**Supplementary Figures S5.Comparison of tumor-infiltrating immune-cell fraction between the high- and low-risk groups by xCELL.**
